# Reducing Problematic Parenting Behaviors, Child Neglect, and Internalizing and Externalizing Problems in Multisystemic Therapy for Child Abuse and Neglect

**DOI:** 10.1177/10775595251381267

**Published:** 2025-09-21

**Authors:** Tom Kirsch, Simone Munsch, Andrea Meyer, Marc Schmid

**Affiliations:** 1 Department of Child and Adolescent Psychiatry, Psychiatric University Clinics Basel, 27209 University of Basel, Basel, Switzerland; 2 Department of Clinical Psychology and Psychotherapy, 27211 University of Fribourg, Fribourg, Switzerland; 3 Department of Clinical Psychology and Epidemiology, Institute of Psychology, 27209 University of Basel, Basel, Switzerland

**Keywords:** multisystemic therapy for child abuse and neglect, child neglect, neglectful parenting, psychological control, internalizing and externalizing problems

## Abstract

Multisystemic Therapy for Child Abuse and Neglect (MST-CAN) has been shown to effectively reduce social worker-assessed child neglect and child problems. However, no research has examined the effects of MST-CAN on parenting behaviors or identified which intervention targets are associated with reductions in child problems. This study examined changes in child internalizing and externalizing problems, parental psychological control, neglectful parenting, and social worker-assessed neglect, accounting for therapist effects, and assessed how parenting and neglect predict child outcomes in 143 parent-child dyads in Switzerland (mean child age = 10.5 years, 54.1% boys). Multilevel regression showed significant reductions in social worker-assessed neglect (b = 14.10, SE = 3.49, *p* < .001) and child problems (b = 4.97*, SE = 0.88, p* < .001) with low intraclass correlation coefficients (ICC = .049, ICC = .017). Neglectful parenting (b = 0.03, SE = 0.05, *p* = .640) and psychological control (b = 0.10, SE = 0.07, *p* = .140) were not significantly reduced. Parenting and social worker assessed neglect did not affect changes in child problems. Findings demonstrate MST-CAN’s effectiveness in reducing social worker-assessed neglect and child problems but highlight the need for targeting psychological control and multi-method and multi-informant assessments of parenting behaviors.

## Introduction

Multisystemic Therapy for Child Abuse and Neglect (MST-CAN; Swenson et al., 2010) aims to decrease child neglect and internalizing and externalizing problems in children by targeting parenting behaviors and social worker–assessed child neglect. MST-CAN has been demonstrated to effectively reduce child problems and social worker-assessed child neglect. However, how MST-CAN specifically addresses parenting behaviors remains unclear. This is important because the link between parenting behaviors and the mental health of children and adolescents is well documented (Pinquart, 2017a, 2017b). In particular, psychological control and neglectful parenting are relevant within the MST-CAN framework. Although neglectful parenting is addressed by MST-CAN, its effectiveness in reducing this behavior has not been empirically examined. Similarly, psychological control, identified as the key predictor of youth mental health outcomes among parenting behaviors (Manuele et al., 2023; Pinquart, 2017a, 2017b; Yan et al., 2020), has not yet been examined in MST-CAN research. Lastly, while MST-CAN aims to reduce neglectful parenting and social worker–assessed child neglect to improve child outcomes, the predictors of treatment success regarding child outcomes in MST-CAN have yet to be empirically identified.

### The Relevance of Child Neglect

Research explicitly focusing on child neglect in the context of child maltreatment remains relatively scarce and was termed the “neglect of neglect” almost four decades ago by Wolock and Horowitz (1984). Neglect is defined as the failure of a child’s caregiver to meet the child’s needs in one or more of the following areas: health, education, emotional development, nutrition, shelter, or safe living conditions (Dubowitz, 2009).

Child neglect stands out as one of the most prevalent forms of child maltreatment (Stoltenborgh et al., 2015). Studies and reports on child neglect prevalence in the U.S., Switzerland, and Germany indicate that self-reported 12-month prevalence rates range from about 4% to 6%, while lifetime prevalence ranges from 7% to 18% (Finkelhor et al., 2015; Lätsch & Stauffer, 2016; Vanderminden et al., 2019; Witt et al., 2017). In contrast, administrative data show lower rates across countries, with 2.6 to 4.7 per 1,000 children identified as having experienced neglect (U.S. Department of Health & Human Services, 2025). In the U.S. in 2023, 7.4 per 1,000 children were identified as having experienced maltreatment, with neglect accounting for 64.1% of cases (4.7 per 1,000) (U.S. Department of Health & Human Services, 2025). National surveys in the U.S. indicate self-reported neglect rates of 5%–6% in the past year and 15%–18% throughout the lifespan (Finkelhor et al., 2015; Vanderminden et al., 2019). Data from Switzerland show that 0.66% of all children were referred to Child Protective Services (CPS) in 2016, with an estimated rate of 2.7% if reporting patterns remained stable (Jud et al., 2021). Of these cases, 15.2% were classified as neglect, although 68% were not categorized into specific forms of maltreatment. For Switzerland, a representative study found that about 4% of adolescents reported neglect in the past year (Lätsch & Stauffer, 2016). In Germany in 2023, CPS substantiated child maltreatment at a rate of 4.5 per 1,000 children, of which 58% were due to neglect (2.6 per 1,000). Self-reports from a representative study in Germany indicated lifetime prevalence rates of 7.1% for emotional neglect and 9.0% for physical neglect (Witt et al., 2017). However, cross-country comparisons must be interpreted with caution due to differences in methodology, reporting systems, and measurement instruments.

Child neglect has been associated with psychopathology and a wide range of cognitive, neurobiological and health problems in both youths and adults (Francis et al., 2023; Jackson et al., 2022; Sand et al., 2024). Meta-analyses have revealed associations between child neglect and higher levels of psychopathology (Francis et al., 2023), depressive symptoms and disorders (Humphreys et al., 2020; Infurna et al., 2016) and an increased risk of anxiety disorders in adolescents and adults (Norman et al., 2012).

### Social Worker Assessed Child Neglect: Associations With Internalizing and Externalizing Problems

Besides self-reports, third-party social worker assessments are crucial in the field of child neglect. The social worker assessment of child neglect used in this study—recognized as one of the best-validated tools in a recent systematic review (Haworth et al., 2024)—defines neglect as the failure of a child’s caregiver to meet the child’s needs in one or more of the following areas: health, education, emotional development, nutrition, shelter, or safe living conditions. Social worker–assessed child neglect captures forms of neglect that are underreported in self-reports (Font & Maguire-Jack, 2020). Moreover, social workers, by their clinical expertise in child protection, are better positioned than other third-party informants to assess neglect, as they use standardized assessment tools across multiple domains and draw on information from various sources to inform their evaluations (Haworth et al., 2024). However, social worker-assessed neglect has been included in only four observational studies examining its relationship with internalizing and externalizing problems. Here, social workers’ assessments of neglect predicted externalizing disorders in children and adolescents in a German sample (Schlensog-Schuster et al., 2022). Two validation studies of child neglect measures have reported an association between social worker-assessed neglect and externalizing behavioral problems (Calheiros et al., 2021; Dubowitz et al., 2005). In a recent study, social workers’ reports of psychological and physical neglect were associated with parents’ reports of their children’s externalizing but not internalizing problems (Silva & Calheiros, 2020).

### Neglectful Parenting and Parental Psychological Control: Associations With Internalizing and Externalizing Problems

In contrast to social worker assessments of child neglect, the relevance of problematic parenting behaviors in various mental health outcomes in youths has been well documented (Manuele et al., 2023; Pinquart, 2017a, 2017b). Meta-analyses have shown associations between parenting behaviors and internalizing and externalizing symptoms (Manuele et al., 2023; Pinquart, 2016, 2017a, 2017b) and child anxiety (McLeod et al., 2007). Parenting interventions have been shown to reduce internalizing problems (Jewell et al., 2022; Sanders et al., 2014; Yap et al., 2016) and externalizing problems (Mingebach et al., 2018; Sanders et al., 2014). However, neglectful parenting and its association with internalizing and externalizing problems remain particularly relevant and are so far understudied.

Neglectful parenting, defined as low behavioral control and a lack of warmth and affection in two relevant meta-analyses (Pinquart, 2017a, 2017b), has been linked to both internalizing and externalizing problems in children (Pinquart, 2017a, 2017b). However, this concept has not been extensively researched, and evidence of its effect is less conclusive than for other parenting methods due to inconsistencies in a limited number of studies. Behavioral control is defined as parenting behaviours that involve setting clear rules, monitoring and applying consistent consequences. Cross-sectional studies have demonstrated that neglectful parenting is associated with internalizing and externalizing problems, while longitudinal studies have linked it specifically to externalizing problems (Pinquart, 2017a, 2017b). Other meta-analyses found small negative associations with self-esteem (Pinquart & Gerke, 2019b). However, neglectful parenting is the least frequently reported parenting behavior in two meta-analyses (Pinquart, 2017a, 2017b), and more recent meta-analyses have not included this parenting behavior altogether (Manuele et al., 2023).

In addition to neglectful parenting, parental psychological control and its association with internalizing and externalizing problems remain particularly relevant in the context of parenting behavior research and interventions. Four meta-analyses have shown consistent associations with psychopathology (Manuele et al., 2023; Pinquart, 2017a, 2017b; Yan et al., 2020). Parental psychological control is a form of control that seeks to manipulate children’s emotional or psychological experiences and is intrusive of children’s thoughts, feelings, and attachments to parents (Barber et al., 2002; Gaertner et al., 2010). This manipulation is often executed through strategies, such as guilt induction, shaming, conditional affection, or withdrawal of affection (Barber et al., 2002). Two meta-analyses showed that psychological control has the strongest association with internalizing and externalizing problems in cross-sectional studies, and exhibits the greatest predictive value in longitudinal studies of all examined parenting behaviors (Pinquart, 2017a, 2017b).

### Multisystemic Therapy for Child Abuse and Neglect

In the field of child protection interventions, MST-CAN represents a standardized, evidence-based, and manualized intervention program designed for families with children and adolescents aged 6–17 years who have experienced abuse or neglect (Swenson et al., 2010). The program has been shown to effectively reduce symptoms of post-traumatic stress disorder and depression in a Randomized Controlled Trial (RCT) in the United States (Swenson et al., 2010). Two studies demonstrated reductions in child psychopathology and decreases in social worker-assessed child neglect throughout MST-CAN (Buderer et al., 2020, 2024), while one study found reductions in parental stress and improvements in parental mental health (Bauch et al., 2022).

Although MST-CAN targets harmful parental practices such as neglectful parenting, and several key components of the intervention address this issue, its effectiveness in this area has not been empirically demonstrated. MST-CAN systematically addresses risk factors for neglectful parenting and social worker–assessed child neglect across multiple ecological levels, including the child, parent, family, and broader community (e.g., school, sports coaches). This ecological perspective is essential, as neglect is a multicausal phenomenon (Swenson & Schaeffer, 2018). MST-CAN places emphasis on the parental level and incorporates CBT-based approaches, including communication and problem-solving skills training, the appropriate use of rules and consequences (behavioral control). These interventions are delivered at high frequency within the family’s natural environment, which enhances adaptability and compliance and is considered central to reducing neglectful parenting and social worker–assessed neglect (Swenson & Schaeffer, 2018). Another potential mechanism of change involves addressing parental trauma symptoms and substance abuse. These are particularly prevalent in the MST-CAN population and are targeted through trauma-focused CBT or reinforcement-based treatment for parents. Mental health problems of parents are among the strongest predictors of child neglect (Mulder et al., 2018). Only one study investigating MST Building Stronger Families (MST-BSF) focused on neglectful parenting. In an RCT, MST-BSF significantly reduced child-reported neglectful parenting (Schaeffer et al., 2021). However, since MST-BSF uses different therapeutic approaches and target different populations than MST-CAN, the results of these studies should not be generalized to MST-CAN. MST-BSF specializes in families where parental substance misuse is present and severe.

While MST-CAN emphasizes promoting appropriate behavioral control and reducing harmful parenting practices (Swenson & Schaeffer, 2018), the role of psychological control within MST-CAN has not been examined. So far, only the impact of the MST-Standard on psychological control has been examined. In one RCT, the MST Standard did not reduce adolescents’ reports of parental psychological control (Asscher et al., 2013). Another study from the Netherlands also did not observe a reduction in psychological control through MST Standard intervention compared to treatment as usual (Deković et al., 2012). As with MST-BSF, the results of MST-Standard cannot be generalized to MST-CAN, as MST-Standard is a specialized intervention designed for youths with severe antisocial behavior.

While studies investigating predictors of child-centered outcomes in MST-CAN are currently lacking, two studies within the context of MST-Standard have addressed this question. Low internalizing problems, reduced substance use, therapist adherence, and therapist experience were identified as predictors of positive child outcomes, including decreased antisocial behavior, continued school attendance, and remaining at home (Lange et al., 2017; Tiernan et al., 2015).

### Therapist-Related Variability in MST-CAN Outcomes

Because MST-CAN is a standardized, manual approach applied by different therapists, the variability in the outcomes related to the treating therapist should be considered. However, no previous MST-CAN studies have accounted for this clustering in their analyses. Only in MST-Standard therapist adherence has been shown to predict improvement in youth behavior and significantly lower rates of youth criminal charges (Schoenwald et al., 2009). Taking therapist variability into account is relevant, as the existing literature shows that therapist effects account for approximately 5% of the variance in therapy outcomes across all studies and 8% in RCTs (Johns et al., 2019). Accounting for variability in treatment outcomes due to different therapists helps prevent overemphasizing the role of the specific treatment model and reduces potential confounding by therapist-related factors (Johns et al., 2019).

### Current Study

This study addresses several gaps in the literature. Although neglectful parenting is a key concept within MST-CAN, prior studies have not examined whether the intervention reduces neglectful parenting. The current study addresses this gap by evaluating the effectiveness of MST-CAN in reducing neglectful parenting. We hypothesize that neglectful parenting will be significantly reduced throughout MST-CAN, given its specific focus on parenting behaviours and child neglect.

Since psychological control is a key correlate of internalizing and externalizing problems in children (Yan et al., 2020), it is important to examine whether parental psychological control is reduced through MST-CAN. Because MST-CAN targets parenting behaviors, aims to reduce harmful parenting practices, and promotes appropriate behavioral control, we hypothesize that parental psychological control will decrease over the course of MST-CAN. We account for differential outcomes across therapists delivering the treatment, as it is well known that there is variability in the effect of standardized treatments across therapists (Johns et al., 2019).

Finally, this study aims to identify predictors of child-centered treatment outcomes in the context of neglectful parenting, social worker-assessed neglect, and psychological control. The first two constructs are directly targeted by MST-CAN and typically occur at high levels in the MST-CAN treatment population, making them especially relevant to examine as potential mechanisms of change. In contrast, psychological control is included to explore its potential relevance for future adaptations of the model, as it is not explicitly addressed in the current version of MST-CAN. To date, no studies have investigated predictors of child-centered outcomes within MST-CAN.

The following hypotheses will be investigated:

**H1:** We expect a reduction from baseline to post-intervention in the following variables while accounting for variability among therapists: 1) neglectful parenting, 2) psychological control 3) social worker assessed child neglect, 4) child’s internalizing and externalizing problems.
**H2:** We expect that reductions and lower levels of children’s internalizing and externalizing problems will be predicted by (1) parental psychological control, (2) neglectful parenting, and (3) social worker–assessed child neglect.

## Methods

### Participants

Study participants were parent–child dyads from families referred to the MST-CAN intervention program in the cantons of Thurgau and Basel-Stadt, Switzerland, between 2015 and 2023. The target child and the parents were invited to participate in the study. The decision regarding which parent would participate was based on the family’s capacitiy and feasibility. For the MST-CAN treatment, children who experienced abuse or neglect at the age of 6–17 years were included. Exclusion criteria were exhibiting suicidal tendencies, homicidal thoughts, psychosis, or a diagnosis of Autism Spectrum Disorder of the target child, as well as ongoing instances of sexual abuse, severe domestic violence, and parental psychosis. Families whose child’s safety was endangered were referred to other types of child protection interventions or measures by CPS. Although the MST-CAN data collection in Switzerland began in 2011, the parenting behavior measures relevant to our study were only introduced in 2015. Therefore, only families referred from 2015 onward were included in the current study and sample. A total of 287 families were referred to MST-CAN between April 2015 and December 2022 by CPS. Of these, 211 families initiated treatment. The remaining 76 families (26.5%) did not begin MST-CAN, primarily due to caregiver refusal (*n* = 32; 11.1%). Additional reasons for non-initiation included referral to alternative services such as sociopedagogical family support (*n* = 2; 0.7%) or other treatment services (*n* = 13; 4.5%), out-of-home placement (*n* = 2; 0.7%), lack of funding (*n* = 1; 0.3%), family relocation (*n* = 2; 0.7%), and not meeting inclusion criteria (*n* = 5; 1.7%). In 19 cases (6.6%), the reason for non-initiation was not specified. Of the 211 families, all were contacted and invited to participate, of whom 164 provided informed consent and were considered eligible for study participation. During the intervention, 21 parent–child dyads (12.8%) dropped out of MST-CAN and were excluded from the study. Reasons for dropout included lack of engagement (*n* = 13; 7.9%), out-of-home care placement (*n* = 6; 3.7%), lack of funding (*n* = 1; 0.6%), and family relocation (*n* = 1; 0.6%). Participants from families who dropped out did not differ significantly from those who completed the intervention with respect to demographic variables (age, sex, and migration background), initial neglect severity, and children’s internalizing and externalizing problems. The final sample included 143 parent–child dyads (see Supplementary Material for participant flow). The number of families per therapist varied, ranging from 1 (0.7%) to 16 (11%) families per therapist, with an average of 6.1 families per therapist. Referrals to MST-CAN were initiated by child welfare agencies or child protection services based on documented events of physical abuse or neglect reported within the preceding 180 days by a social worker.

### Intervention

For a more comprehensive understanding of the treatment content of MST-CAN, we refer to Swenson and Schaeffer (2018) and Swenson et al. (2010). MST-CAN has been implemented in various countries, including the United States, the United Kingdom, Australia, Norway, the Netherlands, and Switzerland. In Switzerland, MST-CAN has been operational since 2011. Due to the high degree of standardization and manualization required by MST Services, the structure and implementation of MST-CAN are consistent across all international settings. Standardization is maintained through licensed agreements regulated by MST Services and reinforced by weekly consultations with MST-CAN experts based in the United States.

MST-CAN addresses families with children at a high risk of abuse and neglect. It combines evidence-based systemic and cognitive-behavioral approaches, guided by Bronfenbrenner (1979) social ecological model, with a case-management approach in the home environment. Families receive two to three sessions per week for up to nine months, starting with diagnostic assessments to identify problem behaviors and risk factors for abuse using various questionnaires and behavioral observations. Since 2022, diagnostic interviews for mental disorders have included children, conducted by psychologists in collaboration with experts in the MST-CAN. After assessment, evidence-based therapies such as cognitive behavioral therapy and trauma therapy are utilized, with pharmacotherapy available if needed. Families also receive 24/7 crisis support. The treatment teams consisted of two psychologists, one psychotherapist, and one psychologist. Both psychologists, holding a master’s degree in clinical psychology and psychotherapists with completed 4–5 years federally licensed post-graduate training in psychotherapy, were engaged in delivering MST-CAN. Ongoing training and weekly consultations ensure treatment fidelity of the therapist, which is assessed with the Therapist Adherence Measure for Child Abuse and Neglect-Revised (TAM-CAN-R).

### Data Collection

This study obtained ethical approval from the local ethics committees (Ethikkommission Ostschweiz, Ethikkommission Nordwest- und Zentralschweiz). Data collection for the study began in July 2011 in the Swiss canton of Thurgau and in November 2014 in the Swiss canton of Basel. The data used in this study were drawn from an ongoing, continuous data collection effort within a naturalistic study context, involving families receiving MST-CAN treatment in Switzerland from April 2015 until December 2022. Before data collection, minors and their legal guardians obtained oral and written informed consent. Parents and children completed questionnaires at the start of treatment and after the intervention, which a research assistant facilitated. Child neglect was evaluated by a social worker from the referring child welfare agency or child protection service, conducted at the beginning and end of the MST-CAN. The caseworker provided information on the case characteristics and type and severity of child neglect experienced by the children.

## Measures

### Internalizing and Externalizing Problems in Youth

To evaluate internalizing and externalizing problems in youth*s,* we used the Child Behavior Checklist (CBCL/4-18; Achenbach, 1991; Workgroup German Version of the Child Behavior Checklist, 1998), which consists of 113 items. Parents rated specific behaviors observed in their child on a three-point scale: “not true” (0), “somewhat or sometimes true” (1), or “always or often true” (2). These items were categorized into eight subscales (social withdrawal; somatic complaints; anxious/depressed; social problems, thought problems, attention problems; and delinquent behaviour and aggressive behavior), along with two broader scales (internalizing and externalizing) and a total score. The raw scores were converted into T-scores for clinical interpretation. In the present sample, the internal consistency for the total score was excellent, with Cronbach’s *α* = 0.93 for the baseline and 0.94 for the postintervention measures.

### Neglectful Parenting and Psychological Control

We used the Zurich Brief Questionnaire for the Assessment of Parental Behaviors to assess reports of the parenting behaviors of psychological control and neglectful parenting (ZKE; Reitzle et al., 2011). Owing to a 70% missing value rate in the child’s version, only the parents’ version was utilized, completed by mothers or fathers. While the children’s version has been validated, the parents’ version has not (Reitzle et al., 2011). The ZKE consists of 32 items rated from “not true” (0) to “always true” (3). As defined in three meta-analyses (Pinquart, 2017a, 2017b; Pinquart & Gerke, 2019a), neglectful parenting combines warmth or support and behavioral control. The items of the ZKE measuring parental warmth and behavioral control were thus recoded to create the “Neglectful Parenting” variable, so that higher values indicate higher neglect (Reitzle et al., 2011). A reduction in neglectful parenting, as defined in this study, thereby reflects an increase in positive parenting behaviors. Psychological control had an acceptable internal consistency of 0.72 at Baseline 1 and 0.73 postintervention, while neglectful parenting showed coefficients of 0.79 at baseline and 0.76 post-intervention.

### Social Worker-Assessed Child Neglect

The Ontario Child Neglect Index (CNI; Trocmé, 1996) assessed child neglect, focusing on six items: supervision, nutrition, clothing and hygiene, physical healthcare, mental healthcare, and developmental and educational care. Trained social workers rated these from “adequate” to “seriously inadequate.” To calculate the total score reflecting the severity of neglect, the highest score among the six scales was combined with an age-related score ranging from 0 to 20. A total score ranging from 0 to 80 reflects the severity of neglect, with higher scores indicating more severe neglect. The age score was added to the highest item score. The German version of the CNI (Pérez et al., 2017) was used, with strong test-retest reliability (weighted kappa 0.83–0.91) and interrater reliability (Pearson’s correlation 0.88–0.91). Significant correlations with other neglect scales supported concurrent validity. Cronbach’s α could not be calculated based on the questionnaire design. Although not directly included as a predictor variable, the interdependence in the data arising from multiple patients being treated by the same therapist was addressed by specifying therapists as random effects in the multilevel models.

### Statistical Analysis

The analyses were conducted using a multilevel regression model, accounting for the hierarchical structure inherent in the data nested within the groups of therapists. The main analyses were performed using the lme4 package in the R statistics software (version 4.3.2, R Core Team, 2023). In testing the first hypothesis, four multilevel regression models were fitted, using change scores for the outcomes of neglectful parenting, social worker-assessed child neglect, children’s internalizing and externalizing problems, and parental psychological control. Parent–child dyads were modelled as Level 1 units and therapists as Level 2 units. The change scores were calculated as difference scores, meaning that the pre-treatment scores were subtracted from the post-treatment scores. For the second hypothesis, a multilevel model was used to examine how changes in internalizing and externalizing problems were associated with various predictors of parenting behavior measured at baseline. We fitted one model containing all predictors and used the change in scores of child problems as the dependent variable (*model 1*). To address potential methodological concerns in the analysis of change scores as used in Model 1 (Tennant et al., 2022) an additional model was fitted containing the same predictors but using the postintervention value of child problems as an outcome, plus the respective pre-intervention value of child problems as a covariate (termed *model* 2). The items linked to positive outcomes reflecting parental warmth and control were recoded to create the “Neglectful Parenting” variable, where higher values indicate more significant neglect. We assessed the normal distribution and independence of the residuals for each model by examining histograms and Q-Q plots. We included the child’s age and assessment period to account for confounding variables. Mean imputation was used for individual questionnaire items for missing data, as Newman (2014) recommended. After mean imputation for baseline measures, missing values ranged from 6.85% for parental psychological control and neglectful parenting to 15.07% for social worker-assessed neglect. Post-intervention measures showed more significant missing values, up to 31.51% for neglectful parenting. A comprehensive approach to multiple imputations was performed using the mice package before data analysis. While multilevel-data-specific methods like 2l.pan were considered, they produced implausible imputed data and exhibited a lower convergence than the predictive mean matching method. The predictive mean matching method yielded plausible values and demonstrated robust convergence after 40 iterations across 20 imputed datasets. Imputation diagnostics involve plotting convergence trends, density plots, and strip plots to ensure the quality and reliability of the imputation process.

## Results

The variables in Table 1 are presented as mean (*M*) and standard deviation (*SD*) for continuous data and proportions for categorical data. The average age was 10.46 years (*SD* = 3.48), and that of the parents was 41.75 years (*SD* = 9.15). The mean treatment duration was 265.9 weeks (*SD* = 31.6). Most children were boys (54.1%), and almost two-thirds (63.0%) of participants were mothers. The average social worker-assessed child neglect values were 37.2 (*SD* = 21.1) at baseline and 24.0 (*SD* = 19.3) postintervention. Average neglectful parenting was 0.67 (*SD* = 0.38) at baseline and 0.63 (*SD* = 0.39) postintervention. The average parental psychological control was 0.95 (*SD* = 0.52) at baseline and 0.83 (*SD* = 0.49) postintervention. Finally, the average values for children’s internalizing and externalizing problems were 65.5 (*SD* = 9.5) at baseline and 59.7 (*SD* = 10.1) postintervention.

**Table 1.** Socioeconomic and Demographic Characteristics of the Study Sample

Variable	Category	Mean (SD) (*n*)	% (*n*)
Child’s age (years)	—	10.5 (3.5) (143)	—
Parent’s age (years)	—	41.8 (9.2) (139)	—
Case duration (days)	—	265.9 (31.6) (136)	—
Child’s gender	Boys	—	54.1% (*n* = 79)
	Girls	—	39.7% (*n* = 58)
Parent’s gender	Men	—	31.5% (*n* = 46)
	Women	—	63.0% (*n* = 92)
Time category	2015	—	11.6% (*n* = 17)
	2016	—	19.9% (*n* = 29)
	2017	—	15.1% (*n* = 22)
	2018	—	10.3% (*n* = 15)
	2019	—	7.5% (*n* = 11)
	2020	—	9.6% (*n* = 14)
	2021	—	8.2% (*n* = 12)
	2022	—	13.0% (*n* = 19)
	2023	—	2.7% (*n* = 4)

*Note.* T1 = Time 1; T2 = Time 2; M = mean; SD = standard deviation.

Table 2 presents the descriptive statistics for the original and imputed datasets, including the means, standard deviations, and ranges (*n* = 20). The range of values across 20 imputed datasets is shown, with the imputed values closely aligned with or falling within the range of the original values. This similarity suggests that the imputation process preserved the overall data structure, indicating that the imputed data approximate the original data.

**Table 2.** Descriptive Statistics of Original and Imputed Datasets

Variable	Original dataset (M ± SD) (n)	Imputed datasets range (M ± SD) (n)
Social worker-assessed child neglect (T1)	37.2 ± 21.1 (*n* = 124)	36.6–38.7 ± 20.9–21.6 (*n* = 146)
Social worker-assessed child neglect (T2)	24.0 ± 19.3 (*n* = 111)	22.4–24.0 ± 18.5–19.5 (*n* = 146)
Neglectful parenting (T1)	0.67 ± 0.38 (*n* = 136)	0.60–0.62 ± 0.36–0.38 (*n* = 146)
Neglectful parenting (T2)	0.63 ± 0.39 (*n* = 100)	0.50–0.57 ± 0.29–0.32 (*n* = 146)
Parental psychological control (T1)	0.95 ± 0.52 (*n* = 136)	0.94–0.96 ± 0.51–0.52 (*n* = 146)
Parental psychological control (T2)	0.83 ± 0.49 (*n* = 105)	0.83–0.91 ± 0.49–0.53 (*n* = 146)
Internalizing and externalizing problems (T1)	65.5 ± 9.5 (*n* = 136)	65.0–65.5 ± 9.4–9.9 (*n* = 146)
Internalizing and externalizing problems (T2)	59.7 ± 10.1 (*n* = 105)	60.0–60.6 ± 10.0–10.9 (*n* = 146)

*Note.* This table provides descriptive statistics for both the original and imputed datasets. Values are presented as mean (M) with standard deviation (SD) and ranges for the imputed datasets. The imputed dataset included 20 imputations. T1 represents pretreatment, and T2 represents posttreatment. Values for social worker–assessed child neglect range from 0 to 80, neglectful parenting and psychological control range from 0 to 3, internalizing and externalizing problems range from 0 to 100.

## Hypothesis 1

Table 3 presents the results of the first hypothesis. Social worker-assessed child neglect (*b* = −14.10, SE = 3.49, *t* = 4.01, *p* < .001, *d* = 0.67, 95% CI [0.54, 0.78]) and child’s internalizing and externalizing problems (*b* = −4.97, *SE* = 0.88, *t* = 5.67, *p* < .001, *d* = 0.53, 95% CI [0.45, 0.61]) showed a notable and significant decrease between baseline and posttreatment. In contrast, neglectful parenting (*b* = −0.03, *SE* = 0.05, *t* = 0.47, *p* = .639, *d* = 0.12, 95% CI [–0.01, 0.26]) and parental psychological control showed only minor and insignificant changes in the same period (*b* = −0.10, *SE* = 0.07, *t* = 1.50, *p* = .140, *d* = 0.20, 95% CI [0.06, 0.35]), with a low intraclass correlation for therapists (ICC = .058). The ICC values among the therapists were .010, .017, .049, and .058 for neglectful parenting, social worker-assessed child neglect, internalizing and externalizing problems, and parental psychological control, respectively.

**Table 3.** Fixed Effects in Multilevel Regression Models

Outcome variable	Estimate	SE	df	t	*p*
Internalizing and externalizing problems	−4.97	0.88	15.2	5.67	<.001
Social worker-assessed child neglect	−14.19	3.49	7.1	4.01	<.001
Parental psychological control	−0.10	0.07	14.9	0.07	.140
Neglectful parenting	−0.03	0.05	97.0	0.47	.639

*Note.* This table presents the fixed effects coefficients from the multilevel regression models that assess variable changes. The fixed effects represent the average impact of each predictor on the outcome variable, assuming that the effect is consistent across all units or groups. Each row represented a different outcome variable.

## Hypothesis 2

### Model 1

The results for Model 1 are presented in Table 4. None of the parenting behaviors predicted changes in children’s internalizing and externalizing problems between baseline and post-intervention (parental psychological control, *b* = 1.55, *t*(347.72) = 1.20, *p* = .232, *R*^2^ = .015, 95% CI [.002, .032]; neglectful parenting, *b* = −1.78, *t*(1190.18) = −0.97, *p* = .333, *R*^2^ = .007, 95% CI [.000, .017]; social worker–assessed neglect, *b* = −0.02, *t*(1161.41) = −0.77, *p* = .444, *R*^2^ = .007, 95% CI [.000, .017]).

**Table 4.** Fixed Effects for the Multilevel Change Score Model Predicting Post-Intervention Outcomes

Fixed effects	Estimate	SE	df	t	*p*
Neglectful parenting	−1.78	1.83	1190.2	−0.97	.333
Psychological control	1.55	1.29	347.7	1.20	.232
Social worker-assessed child neglect	−0.02	0.03	1161.4	−0.77	.444

*Note.* This table presents fixed-effects estimates from the multilevel change-score model for predicting post-intervention outcomes. The fixed effects represent the average effect of each predictor on the outcome variable.

### Model 2

The results of Model 2 are comparable to those of Model 1 and are presented in Table 5. Thus under this model, none of the predictors of parenting behavior predicted children’s internalizing and externalizing problems postintervention, controlling for children’s internalizing and externalizing problems at baseline (parental psychological control, *b* = 0.04, *t*(344.64) = 0.03, *p* = .980, *R*^2^ = .002, 95% CI [.000, .008]; neglectful parenting, *b* = 2.90, *t*(529.60) = 1.15, *p* = .250, *R*^2^ = .009, 95% CI [.002, .017]; social worker–assessed neglect, *b* = 0.02, *t*(596.87) = 0.70, *p* = .484, *R*^2^ = .006, 95% CI [.001, .021]).

**Table 5.** Effects of the Multilevel Baseline Covariate Model Predicting Post-Intervention Outcomes

Fixed effects	Estimate	SE	df	t	*p*
Neglectful parenting	2.90	1.81	529.6	1.15	.250
Psychological control	0.04	1.37	344.6	0.03	.980
Social worker-assessed child neglect	0.02	0.03	596.9	0.70	.484

*Note.* This table presents the fixed effects estimate from the multilevel baseline covariate model predicting post-intervention outcomes. The fixed effects represent the average effect of each predictor on the outcome variable.

## Discussion

This study aimed to examine whether neglectful parenting, psychological control, social worker-assessed child neglect, and children’s internalizing and externalizing problems decreased from baseline to post-intervention, accounting for therapist variability. Additionally, we explored whether reductions in children’s internalizing and externalizing problems were predicted by baseline levels of neglectful parenting, psychological control, and social worker-assessed child neglect. In our study, we observed that social workerassessed child neglect and internalizing and externalizing problems significantly decreased from pre-to posttreatment during MST-CAN (Hypothesis 1) and thereby replicated the findings of Buderer et al. (2020). The present study found larger effects for the reductions in internalizing and externalizing problems and social worker assessed neglect compared with the RCT by Swenson et al. (2010). Because the effects in the current study appear larger than in the RCT, the effects of this study may be somewhat overestimated. This may be partly due to the absence of a control group, as within-subject effect sizes tend to appear larger. However, the study designs and statistical methods differ substantially, and therefore, direct comparisons should be interpreted with caution.

Notably, this study is the first in the MST-CAN context to account for the interdependence in the data arising from multiple patients being treated by the same therapist. The variability in all four outcomes was much higher among patients than among the therapists. This may be related to the emphasis of MST-CAN on the standardized training of therapists to increase treatment fidelity and adherence (Schoenwald et al., 2009). Contrary to our hypotheses, the levels of psychological control, neglectful parenting, and social worker-assessed child neglect at the beginning of the MST-CAN did not predict reductions in internalizing and externalizing problems after MST-CAN (Hypothesis 2). None of the predictors examined in this study showed greater relevance than others in explaining internalizing or externalizing problems following the MST-CAN (Hypothesis 2). Specifically, the reduction in child internalizing and externalizing symptoms was not significantly influenced by the level of parental self-reported psychological control, neglectful parenting, or social workers’ assessment of child neglect at the beginning of MST-CAN.

The high degree of standardization, quality assurance, and uniform implementation of MST-CAN across international contexts increases the likelihood that our findings from the Swiss MST-CAN sample could be generalizable to other communities. However, this standardization cannot control for the influence of sociocultural factors that differ between countries, meaning that the results should still be replicated in different sociocultural contexts.

Regarding the first hypothesis, we assume that the reduction in social worker–assessed child neglect is driven by MST-CAN’s strong focus on systematically identifying the underlying drivers of neglect, particularly at the parental level. These drivers are addressed through targeted interventions, with a focus on improving parental behavioral control and strengthening parental resources. These interventions are delivered directly in the family’s natural environment—an element considered central to MST-CAN’s effectiveness in reducing child neglect. Research has identified parental mental health problems as a major risk factor for child neglect (Mulder et al., 2018). In a Swiss sample, MST-CAN has demonstrated reductions in psychological distress of parents, which contributed to a reduction in social worker assessed-child neglect (Hefti et al., 2020).

The lack of reduction in psychological control (Hypothesis 1) may, at least in part, be explained by the fact that a focus on improving behavioral control in MST-CAN does not necessarily lead to a reduction in psychologically controlling behaviors. Higher levels of appropriate use of transparent rules, consequences, and monitoring (behavioural control) might not imply lower levels of psychological control (e.g., low levels of guilt induction, invalidation of emotional reactions, mistrust in parents towards their children). Weak negative correlations between behavioral and psychological control, ranging from* r *= −.23 to −.13, were reported in three studies comparing these parenting behaviours (Barber, 1996; Barber et al., 1994, 2002). This aligns with studies examining the effects of the MST-Standard, where psychological control does not represent a primary target in contrast to behavioral control. These two studies investigated adolescent-reported psychological control and found no evidence of reduction during MST-Standard (Asscher et al., 2013; Deković et al., 2012). Both MST-Standard and MST-CAN emphasize an increase in more appropriate behavioral control strategies, including setting rules and boundaries, applying appropriate negative consequences, and using positive reinforcement. Future studies should investigate whether the integration of attachment- and emotion-focused parenting interventions into MST-CAN could reduce parents’ psychological control. Such interventions aim to help parents appropriately recognize and respond to their children’s attachment and emotional needs (Jugovac et al., 2022).

The nonreduction and lack of predictive value of neglectful parenting and psychological control (Hypotheses 1 and 2) may also partly be attributed to social desirability bias in parent reports, which is especially pronounced before the MST-CAN treatment. In our sample, parents from the context of Child Protective Services may have felt pressured to reply more positively due to feelings of shame and guilt (Gibson, 2015) and concerns regarding legal consequences such as child placement or welfare concerns (Cooley & Jackson, 2022). Such response behavior might have resulted in a floor effect, where many parents scored at or near the lowest possible value of the measure, thereby reducing the variability and limiting the predictive power to detect a treatment effect (Šimkovic & Träuble, 2019). This is reflected in our results, where levels of neglectful parenting (*M* = 0.67, *SD* = 0.38) and psychological control (*M* = 0.95, *SD* = 0.52) were already very low at baseline. The relatively small standard deviations (*SD* = 0.38 for neglectful parenting; *SD* = 0.52 for psychological control) indicate a tight clustering of scores around the mean. Consequently, changes throughout treatment were minimal (*b* = −0.03 for neglectful parenting; *b* = −0.10 for psychological control). This assumption aligns with a study validating the MST-Building Stronger Families (BSF), in which parent-reported neglectful parenting was rated zero on all items. Simultaneously, child reports were much higher (Schaeffer et al., 2021). A recent systematic review found that parents in CPS contexts tend to report fewer dysfunctional parenting methods than their children (Cooley & Jackson, 2022).

The floor effect observed in the assessment of parenting behaviors underscores the importance of utilizing multi-informant, multi-method evaluations, particularly in high-risk populations involving different stakeholders. While such approaches have already been employed for child neglect, there is a need to extend these methods to evaluate parenting behaviors to ensure more accurate and sensitive measurements. Additionally, Ecological Momentary Assessment (EMA) represents a promising approach to reduce response bias due to social desirability because it reduces flaws related to retrospective reporting (Mofsen et al., 2019).

Furthermore, to obtain more reliable and valid responses in naturalistic treatment research, it may be beneficial to use the collected data for research and therapeutic purposes. Currently, the data are used solely for research purposes. However, if parents are given a clear rationale on how their reports contribute to their therapeutic process, this could potentially improve their response behavior. A previous study found that patients were more likely to comply when they perceived research as directly benefiting their therapy (Schiepek et al., 2016).

Social worker-assessed child neglect did not predict child outcomes in MST-CAN (Hypothesis 2). This may be due to a missing association between social worker assessed neglect and internalizing problems in youth, while we used internalizing and externalizing symptoms as outcome variables. As shown in three studies, social worker-assessed neglect was primarily linked to observable externalizing behaviors in children (Calheiros et al., 2021; Dubowitz et al., 2005; Silva & Calheiros, 2020). This indicates that neglect might be associated in short-therm with children exhibiting externalizing problems to meet their unmet needs and gain attention. The association between neglect and internalizing problems might further be influenced by longer-term developmental trajectories and the effect on internalizing problems might emerge only later in adolescence and adulthood (VanMeter et al., 2021). In addition, existing research on associations between child neglect and psychopathology usually relies on retrospective reports or involves a time lag of several years between the occurrence of neglect and the manifestation of internalizing and externalizing symptoms (Francis et al., 2023).

Since we did not find any predictive effect of the examined variables (neglectful parenting, psychological control and social worker-assessed child neglect), we assume that MST-CAN may exert direct effects on child outcomes, rather than operating solely through mediation by parenting variables. Child-focused therapeutic components and evidence-based child-focused interventions within MST-CAN, such as behavioral activation and exposure therapy, as well as common factors such as problem-solving support and motivational clarification, are likely to contribute to improvements in child outcomes. Secondly, based on the study by Bauch et al. (2022) documenting improvements in parental stress and parental mental health during MST-CAN, we infer that these improvements might predict positive changes in child internalizing and externalizing problems. Both parental stress and parental mental health are well known risk factors for child internalizing and externalizing problems (Ribas et al., 2024; Stracke et al., 2023).

### Limitations

First, since this is an observational study, we cannot isolate the effect of the intervention and therefore cannot infer that the observed changes were caused by MST-CAN rather than by other variables or events occurring during the treatment (e.g., CPS involvement, changes in social relationships, or financial circumstances). Likewise, we cannot conclude that the absence of reductions in parenting behaviors indicates that MST-CAN was ineffective. Additional sources of bias of our study design must be considered, including regression to the mean, whereby elevated problem behavior may decline naturally over time. Selection bias may also have occurred, as families who agreed to participate may differ systematically from those who received MST-CAN but did not consent, thereby limiting generalizability.

Second, we assessed changes in variables only at two time points over nine months. This design is limited for capturing intervention-related change, as it provides no information on trajectories, patterns, and the timing of changes. It also increases susceptibility to random influences and measurement error. Future research should therefore incorporate more frequent assessments across the intervention period.

Third, parenting behaviors and children’s internalizing problems were assessed solely through parent reports with the support of a research assistant. In addition to the associated floor effect, relying solely on parent reports of children’s internalizing problems raises concerns, as it is well-documented that parents often overlook and underestimate internalizing symptoms in children (Durbeej et al., 2019). Additionally, parents’ mental health can impair their perception of their children’s symptoms (Liskola et al., 2021). This bias may have been particularly pronounced in our high-risk family sample.

Finally, we did not have specific information on the type of social worker-assessed child neglect. While controlling for different forms of neglect could have provided more insights into the predictive value of neglect, our focus was on conceptualizing social worker-assessed neglect as a third-party assessment and comparing it to self-reported parenting behaviors. Additionally, because social workers were involved in referring families to MST-CAN, this may have positively influenced their outcome assessments and may have induced expectancy bias. However, we strongly believe that social workers operating within the MST-CAN child welfare context feel obliged to adhere to their professional and ethical duty to remain objective.

### Future Research

If our results are replicated, they highlight the potential to increase the effects of MST-CAN by including more specific interventions that target psychological control. While particular interventions targeting psychological control are currently underrepresented in the scientific literature, attachment- and emotion-focused parenting programs have gained popularity as effective interventions (Jugovac et al., 2022). Future research in the context of MST-CAN should clarify whether incorporating such approaches can further enhance the effects of MST-CAN. Based on the findings of Bauch et al. (2022), future studies should investigate whether parental stress and parental mental health can predict reductions in children’s internalizing and externalizing problems in MST-CAN. Our findings also underscore the importance of multi-method and multi-informant assessments, particularly in high-risk populations with parent reports. Therefore, EMA studies could be a promising approach for future research to reduce social desirability bias and capture experiences in real-time and in the naturalistic context of MST-CAN. Based on EMA-assessed predictors, future research should focus on which parental or child-related behaviors predict which child–parent dyads are most likely to benefit from MST-CAN. Additionally, we suggest integrating research-based information into the therapeutic process to increase acceptance and relevance of research in naturalistic clinical settings. This might, in turn, reduce underreporting of problematic parenting behaviors.

### Conclusion

This study strengthens the evidence that MST-CAN is effective in reducing social worker-assessed child neglect and internalizing and externalizing problems. However, it also highlights mixed findings in the sense that parents’ self-reports on their parenting in the context of child protection interventions may be biased, leading to nonreduction and missing predictive information, underscoring the need for a multi-method approach. Our results indicate that the relationship between social worker–assessed child neglect and internalizing and externalizing symptoms is complex and appears to have more of a developmental impact over time rather than a strong short-term effect. Finally, MST-CAN may not effectively reduce parental psychological control by primarily targeting improvements in behavioral control and might therefore benefit from the integration of attachment- and emotion-focused parenting interventions.

## Supplemental Material

Supplemental Material - Reducing Problematic Parenting Behaviors, Child Neglect, and Internalizing and Externalizing Problems in Multisystemic Therapy for Child Abuse and Neglect Supplemental Material for Reducing Problematic Parenting Behaviors, Child Neglect, and Internalizing and Externalizing Problems in Multisystemic Therapy for Child Abuse and Neglect by Tom Kirsch, Simone Munsch, Andrea Meyer and Marc Schmid in Child Maltreatment

## Data Availability

Due to data protection regulations mandated by the Ethics Commission, we are unable to share the raw data. The dataset contains highly sensitive patient information, and its confidentiality must be strictly maintained.*
