# Supplementary material for: Reducing Problematic Parenting Behaviors, Child Neglect, and Internalizing and Externalizing Problems in Multisystemic Therapy for Child Abuse and Neglect
Source: Child Maltreat. 2025 Sep 21;31(3):515–28. doi: 10.1177/10775595251381267 (PMC13264655; doi:10.1177/10775595251381267)
Supplement: Supplemental Material - Reducing Problematic Parenting Behaviors, Child Neglect, and Internalizing and Externalizing Problems in Multisystemic Therapy for Child Abuse and Neglect [file sj-pdf-1-cmx-10.1177_10775595251381267.pdf]

Supplementary Material A. CONSORT Flow Diagram of Participant Progress

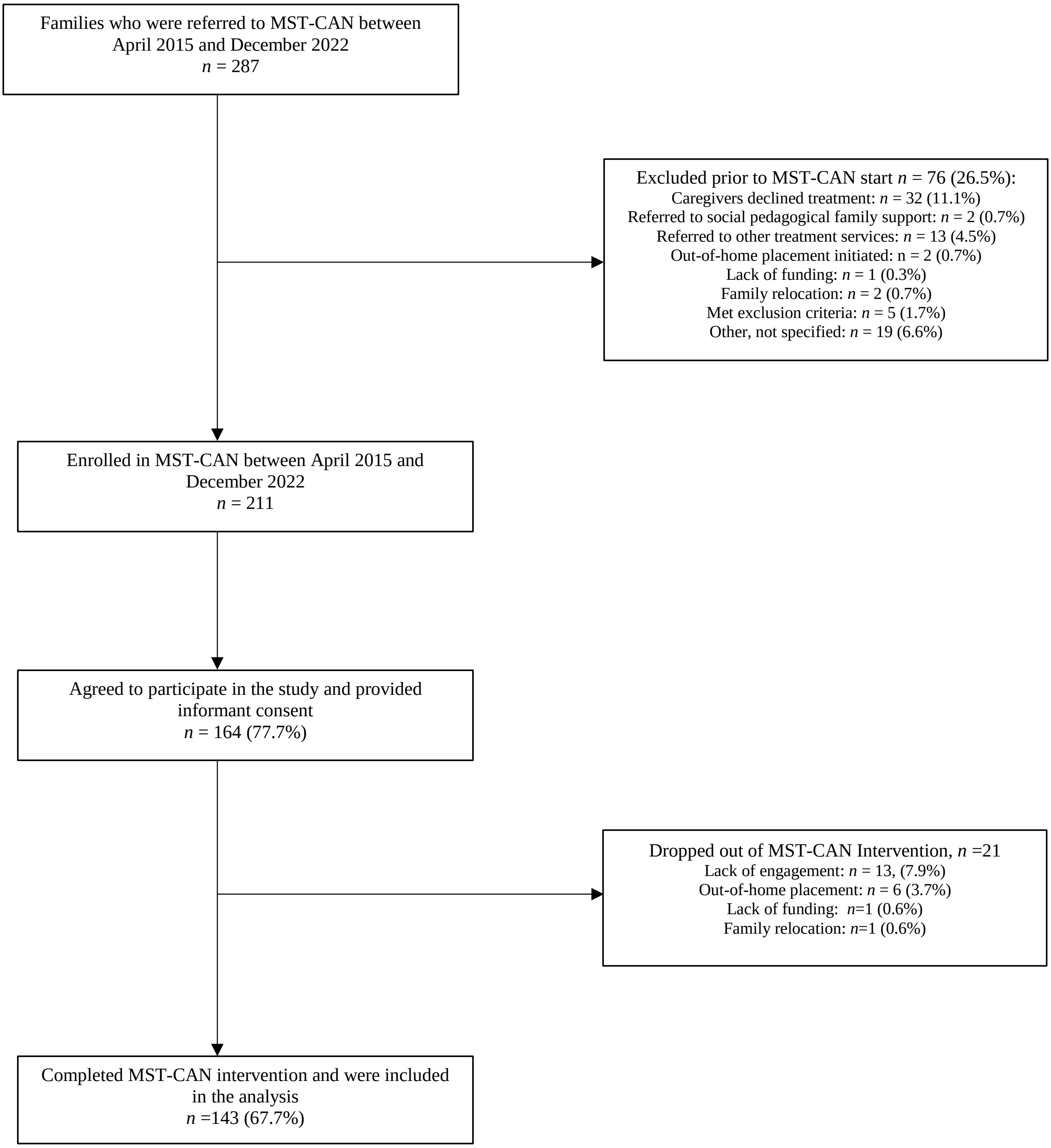

Note. Participants who dropped out of the intervention (n = 21) were excluded from all analyses, regardless of whether baseline data were available. Dropout percentages are calculated relative to the 164 participants who provided informed consent and began the intervention. “Completer” refers to termination of treatment, not necessarily the full completion of all assessments.
